# Carbon and Nitrogen Burial and Response to Climate Change and Anthropogenic Disturbance in Chaohu Lake, China

**DOI:** 10.3390/ijerph15122734

**Published:** 2018-12-04

**Authors:** Qibiao Yu, Fang Wang, Weijin Yan, Fengsong Zhang, Shucong Lv, Yanqiang Li

**Affiliations:** 1 Institute of Geographic Sciences and Natural Resources Research, Chinese Academy of Sciences, Beijing 100101, China; yuqibiao2007@126.com (Q.Y.); wangf@igsnrr.ac.cn (F.W.); lvsc.16b@igsnrr.ac.cn (S.L.); leeyqi@163.com (Y.L.); 2 University of Chinese Academy of Sciences, Beijing 100049, China

**Keywords:** Chaohu Lake, carbon and nitrogen, burial rate, δ^13^C and δ^15^N values, C/N, climate change

## Abstract

Lakes are a crucial component of the global carbon and nitrogen cycle. As a trend of enhanced human activities and climate change, the mechanisms of burial remain poorly understood. In this study, diverse biogeochemical techniques were applied to analyze the temporal variation of organic carbon and nitrogen burial rates in Chaohu Lake. The results showed that burial rates have ranged from 9.39 to 35.87 g C m^−2^ yr^−1^ for carbon and from 1.66 to 5.67 g N m^−2^ yr^−1^ for nitrogen since the 1860s. The average rates were 19.6 g C m^−2^ yr^−1^ and 3.14 g N m^−2^ yr^−1^ after the 1970s, which were significantly higher than the rate before the 1970s, showing an increasing trend. The decrease of C/N ratios as well as organic matter δ^13^C values indicates that the major organic matter source in sediment has been algal production since the 1970s. The increase of δ^15^N values indicated that the promotion in productivity was stimulated by nutrient input from sewage and agricultural runoff. The burial rates of organic carbon and nitrogen were significantly positively related to socio-economics and temperature, indicating that Chaohu Lake will become an increasing carbon and nitrogen pool under conditions of enhanced human activities and intensive precipitation.

## 1. Introduction

Lakes are hotspots for the carbon (C) and nitrogen (N) biogeochemical cycle due to their production, transformation, and transportation [1,2,3,4]. Lake sediments bury a considerable amount of organic carbon and nitrogen and are considered to be of vital importance in global carbon and nitrogen sequestration [5,6,7,8,9,10,11], with an estimation of 0.6 × 10^15^ g C yr^−1^ for carbon [8] and 4.93 to 49.3 4.93 to 49.3 × 10^12^ N yr^−1^ for nitrogen [12]. Many studies have revealed that organic carbon burial rates in the sediment of lakes have increased in the last 100–150 years [7,9,13,14]. Human activities and climate change have been recognized as the primary driving factors that have a significant influence on the carbon and nitrogen cycle in lakes [15,16,17,18,19]. During the last century, nutrients from industrial wastewater, domestic sewage, and agricultural runoff have increased substantially [20,21]. The enrichment of nutrients has remarkable effects on the primary production and ecological structure in lakes [22]. Increasing temperature can stimulate algal growth and lead to the increase of primary production [23], but it can also accelerate decomposition and decrease the burial efficiency of organic matter after sedimentation [15]. Precipitation has no direct influence on the burial rates in lakes. However, changes in precipitation patterns along with enhanced nutrient loading can promote nutrient import from terrestrial ecosystems [24,25] and further raise productivity in lakes and organic matter burial rates in sediment.

Carbon and nitrogen buried in sediment is a mixture between that originating from terrestrial ecosystems (termed as an allochthonous source) and that produced by primary production in lakes (termed as an autochthonous source) [8]. Allochthonous source input is controlled by human activities such as deforestation, urbanization, and the construction of reservoirs [13]. Allochthonous sources are related to productivity in lakes, which are affected by nutrient loading [14]. Therefore, identifying the changes of organic matter sources in sediment is important in understanding the historical change in the trophic status of a lake and its driving factors. Geochemical proxies such as the C/N ratio and the δ^13^C and δ^15^N values of organic matter in sediment can provide reliable paleo-environmental information [2,26] and have been widely used to identify organic matter sources in lakes. Different sources of organic matter are characterized by different C/N ratios. Autochthonous organic sources, such as algae, are marked by high protein content and low ratios of C/N, ranging from 3 to 8 [27]. Allochthonous sources, such as C3 and C4 land plants, are characterized by an abundance of cellulose and high C/N ratios (>20) [2]. The range of carbon isotopic composition in organic matter shows an overlap between various sources where the typical range of δ^13^C values is −30‰ to −20‰ for algae in lake, −32‰ to −20‰ for C3 land plants and −15‰ to −10‰ for C4 land plants [28]. Even though δ^13^C values overlap, the different δ^13^C values of C3 land plants can be combined and used with the C/N ratios to assess the relative contribution of organic matter sources in sediment from lake-derived and terrestrial organic matter [29]. The δ^15^N value is also a widely used proxy to identify the sources of organic matter in sediment because of the difference in nitrogen availability for terrestrial plants and in-lake phytoplankton. Terrestrial plants that utilize nitrogen fixed by N_2_-fixer in soil are characterized by low δ^15^N values, with a mean value of 0‰ [28]. N_2_-fixing cyanobacteria in lakes using N_2_ from the atmosphere have a similar value of 0‰ [30]. Nitrate is the most commonly utilized form of dissolved inorganic nitrogen (DIN) for non-N_2_ fixing algae, which is mobile and easily exported from the terrestrial landscape. Nitrate originating from agricultural runoff and animal sewage are marked by typical isotopically heavy nitrogen (δ^15^N = +10 to +25‰) [31], which can elevate δ^15^N values in algae and organic matter. However, few studies have simultaneously utilized all three proxies to identify carbon and nitrogen burial variation and source changes in lake sediment and further provide a robust description of the driving factors on burial rates from climate change and human activities.

Chaohu Lake, located in southeast China, is a typical shallow lake located in the lower reach of the Changjiang River. In the last century, the water quality, trophic level, and ecological structure in the lake has experienced a dramatic change under enhanced human activities such as deforestation, changing of the tillage system, urbanization, and damming [32,33,34]. The temporal change of nutrient sources from the catchment and the ecological structure of the lake are well understood [35,36,37], but variations in carbon and nitrogen burial and the driving factors from human activity and climate change remain unclear. In this study, we investigated the temporal variability of carbon and nitrogen burial rates from two sediment cores in Chaohu Lake. The main objectives of this study were: (1) to investigate the temporal variability of carbon and nitrogen burial rates of Chaohu Lake, (2) to identify the change of organic matter sources, and (3) to analyze the driving factors for carbon and nitrogen burial rates.

## 2. Materials and Methods

### 2.1. Study Area

**Figure 1:**
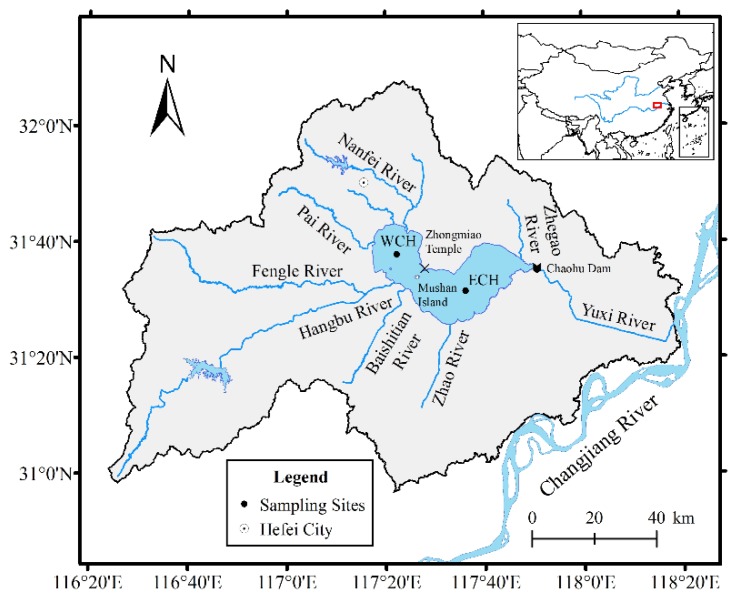
Location of Chaohu Lake and the sampling site in eastern Chaohu Lake (ECH) and western Chaohu Lake (WCH).

Chaohu Lake (30°59′ N–32°06′ N, 116°24′ E–118°00′ E) is one of the five largest freshwater lakes in China and is located in the lower reaches of the Changjiang River (Figure 1). The lake has a catchment area of 13,350 km^2^ and a surface area of 783 km^2^. The entire watershed is located in the temperate monsoon climate zone, with an annual mean temperature of 15.5 °C and precipitation of 1000 mm. Chaohu Lake can be divided into two regions by a line crossing Zhongmiao Temple and Mushan Island: the eastern lake (about one third of its surface area) and western lake (about two thirds of its surface area). The Nanfei River and Hangbu River, covering more than 60% of the entire watershed, are the two largest rivers that discharge into the western lake. The Yuxi River is the only outflow river linking to the Changjiang River. Prior to the construction of the Chaohu Dam in 1962, the water of Chaohu Lake could naturally exchange with the water of the Changjiang River. Since then, Chaohu Lake has become a man-controlled reservoir-lake.

### 2.2. Sampling and Analytical Methods

In October 2016, two sediment cores located at the eastern lake (ECH, 117°36′14.38″ E, 31°31′28.92″ N) and western lake (WCH, 117°22′20.49″ E, 31°37′50.59″ N) (Figure 1) were collected using gravity sampling cores that were 28 cm and 29 cm in length, respectively. The general characteristics of these two cores are presented in Table 1. The sediment cores were sliced into 1 cm subsamples in situ and kept frozen by a portative cooler. After being taken to the laboratory, the samples were weighed wet, freeze-dried, then reweighed to calculate the dry bulk density.

Sedimentary chronology was determined using the ^210^Pb radiometric dating method. The radionuclides (^210^Pb, ^226^Ra and ^137^Cs) were measured for 40,000 s (live time) using well-type coaxial gamma ray spectrometers equipped with a high purity germanium detection (GWL-120-10, ORTEC, Oak Ridge, TN, USA) and a digital signal processing gamma ray spectrometer (DSPEC jr 2.0, ORTEC, Oak Ridge, TN, USA). Before testing, each sample was sealed in a container for over three weeks to ensure radioactive equilibration.

Total organic carbon (TOC) was tested by an element analyzer (Vario MICRO cube, Elementar, Langenselbold, Germany) after removing the inorganic carbon by adding HCl. The total nitrogen (TN) of untreated sediment samples was measured by an element analyzer (Vario MICRO cube, Elementar, Langenselbold, Germany). Sediment samples for C and N isotope compositions in organic matter were pretreated with 1 M HCl to remove inorganic carbon, followed by centrifugation, rinsing with deionized water to obtain a neutral pH condition, then dried at 60 °C for 4 h. The isotope ratios were determined by a Finnigan MAT 253 mass spectrometer (Thermo Electron Corporation, Ringoes, NJ, USA) equipped with a Flash EA 1112 elemental analyzer spectrometer (Thermo Electron Corporation, Ringoes, NJ, USA). The isotopic compositions of ^13^C and ^15^N were expressed in δ and expressed as per mill (‰) relative to the standard of Pee Dee Belemnite and atmospheric N_2_ for δ^13^C and δ^15^N, respectively.

### 2.3. Calculation of Carbon and Nitrogen Sedimentation Rates

The sedimentary chronology of the cores was calculated by applying the ^210^Pb constant rate of supply (CRS) model. This model assumes that there is a constant flux of ^210^Pb and a varying flux of sediment supply [38]. Therefore, this model can be used for lakes where the sediment supply varies with changing human activities and climatic change [39,40]. The CRS dating model can be expressed as:

(1) $$t\;=\frac{1}{\lambda }\ln\frac{A_{0}}{A_{h}}$$

where, t is the date of sediment for layer h; A_h_ is is the cumulative inventory of excess ^210^Pb (^210^Pb_ex_ = ^210^Pb − ^226^Ra) below layer t (Bq kg^−1^); A_0_ is the cumulative inventory of excess ^210^Pb for the entire core (Bq kg^−1^); λ is the decay constant of ^210^Pb (0.03114 year^−1^).

However, using the CRS model may cause discrepancies due to the change of the sedimentation environment [38]. In this study, the sedimentary chronology of the cores was calculated by applying the ^210^Pb composite model combined with the peak of ^137^Cs at 1963 as a time marker to validate the sediment chronology [38]. Calculations used the peak layer of ^137^Cs, which corresponds to the year 1963 to divide the sediment core into two sections: below the 1963-layer (pre-1963) and above the 1963-layer (post-1963). For the post-1963 layers, the dates were calculated as:

(2) $$T_{h}{=\;T}_{0}+\;\frac{1}{\lambda }\ln(1\;+\;\lambda \frac{A_{0}{\;-\;A}_{h}}{P})$$

where, T_h_ is the sampling time (25 October 2016); λ is the decay constant of ^210^Pb (0.03114 year^−1^); A_0_ is the cumulative inventory of excess ^210^Pb for the entire core (Bq kg^−1^); A_h_ is the cumulative inventory of excess ^210^Pb below layer h (Bq kg^−1^); and P is the supply rate of excess ^210^Pb, which is calculated as below:

(3) $$P\;=\;\frac{-\lambda (A_{0}{\;-\;A}_{w})}{{1\;-\;e}^{{-\lambda (T}_{0}\;-1963)}}$$

where, A_w_ is the cumulative inventory of excess ^210^Pb below the 1963-layer (Bq kg^−1^).

For the pre-1963 layers, the dates were calculated as below:

(4) $$T_{i}\;=\;1963\;-\frac{1}{\lambda }\ln\frac{A_{w}}{A_{i}}$$

where, A_i_ is the cumulative inventory of excess ^210^Pb below layer i (Bq kg^−1^).

Consequently, the dry mass accumulation rates (DMAR, g cm^−2^ yr^−1^) can be calculated by the following equation:

(5) $$\mathrm{DMAR}\;=\;\rho \frac{\partial z}{\partial t}$$

where, ρ is the dry sediment density (g cm^−3^), z is the depth of the sediment layer (cm); and t is the date of the sediment (yr).

Carbon or nitrogen burial rates (SR, g C m^−2^ yr^−1^ or g N m^−2^ yr^−1^) in sediment can be estimated by a calculation as per the equation below:

(6) SR = DMARφ

where, *φ* is the content of TOC or TN (%).

### 2.4. Data Analysis

The changes in climate and human activities have a direct influence on nutrient export from terrestrial ecosystems. The change in climatic factors such as temperature and precipitation, and social development factors such as damming, chemical fertilizer usage, human population, and economy were analyzed as driving factors for the carbon or nitrogen burial rates. Temperature, precipitation, and wind speed data were collected from national meteorological stations (http://data.cma.cn). The water level and discharge of the Chaohu Dam was culled from Annual Hydrological Report, China (Hydrological Data of Changjiang River Basin). Chemical fertilizer usage, GDP (gross domestic product), and population were calculated from county level data based on an area within the Chaohu Lake watershed, which was collected from the Anhui statistical yearbook (1988–2016, http://tongji.cnki.net) and the local chronicles of Anhui Province (1953–1987, http://www.ahdfz.gov.cn). Correlation coefficients between these factors and organic carbon and nitrogen burial rates were calculated. The combination of principal component analysis (PCA) and multiple linear regression (MLR) were developed to obtain the regression equation between these factors and burial rates. PCA can extract variable information from a set of correlated variables of observations into uncorrelated variables called principal components (PCs), which was performed by the built-in *pca* function in MATLAB software (R2016b, MathWorks, Natick, MA, USA).

## 3. Results

### 3.1. Chronology of Sediment Cores

**Figure 2:**
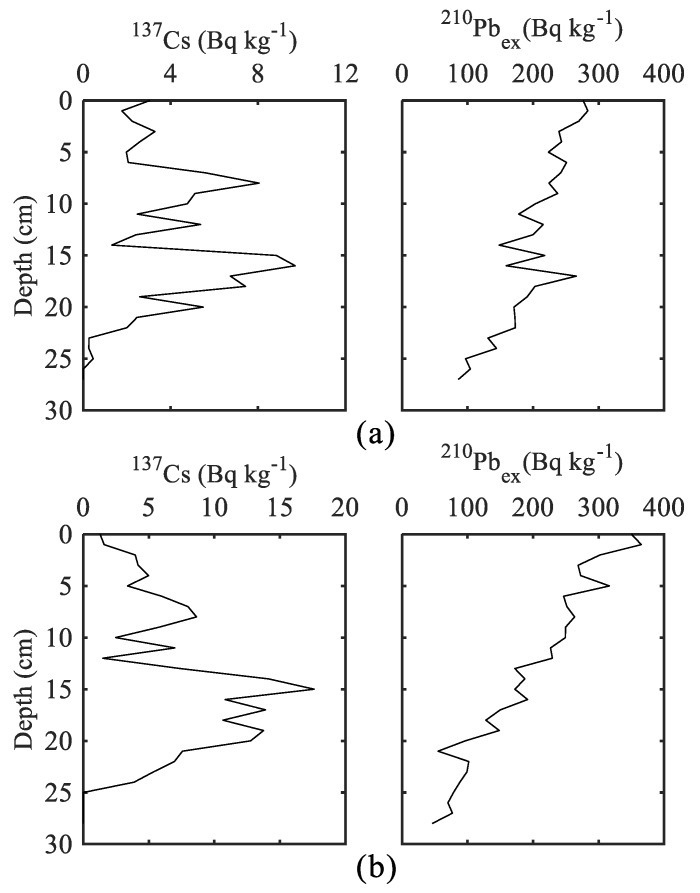
Vertical distributions of ^137^Cs and excess ^210^Pb at (**a**) the ECH and (**b**) WCH.

**Figure 3:**
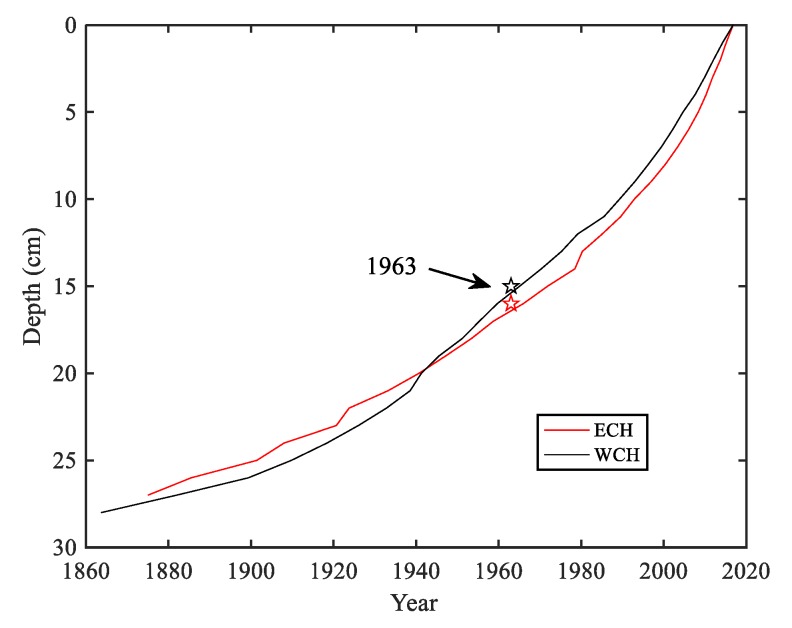
Chronology of cores calculated from the ^137^Cs validated composite model.

The vertical distributions of ^210^Pb_ex_ and ^137^Cs activities of ECH and WCH are shown in Figure 2. The profiles of ^210^Pb_ex_ presented exponential variation, with the highest values in the top layers. The ^137^Cs peaked at 16 cm and 15 cm with 9.70 and 17.62 Bq kg^−1^ in the ECH and WCH cores, respectively, which can be linked to the intensive human nuclear testing [41] and are widely used as time markers to calculate sediment chronology [38,39,40,42]. Combined with the peaks of ^137^Cs, the results of the validated composite model showed that both of the two sediment cores dated to the 1860s and 1870s at the ECH and WCH, respectively (Figure 3).

### 3.2. Geochemical Proxies in Sediment Cores

**Figure 4:**
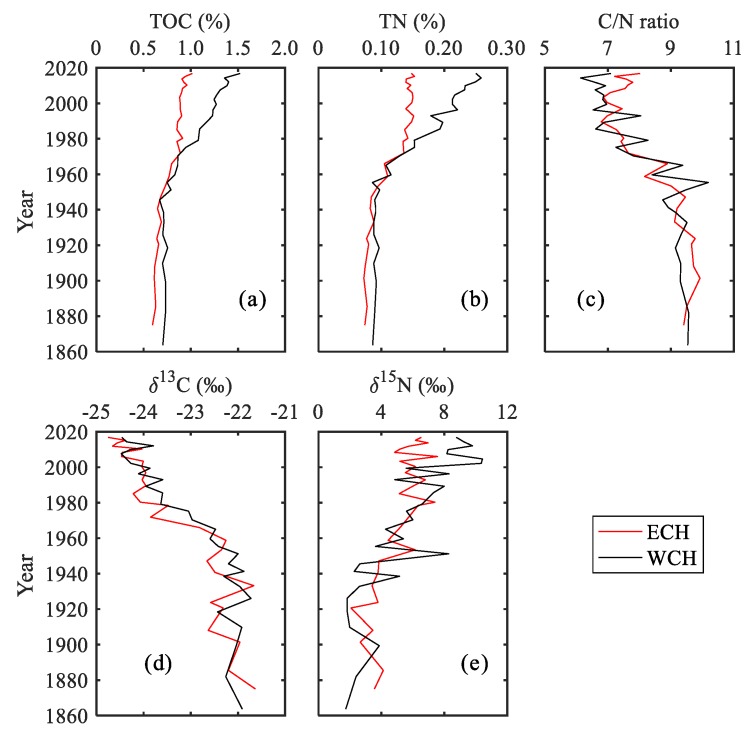
Variations of (**a**) total organic carbon contents (TOC), (**b**) total nitrogen contents (TN), (**c**) C/N ratios, (**d**) δ^13^C values and (**e**) δ^15^ N values in sediment at the ECH and WCH.

The vertical distribution of TOC content ranged from 0.59% to 1.02% (with an average 0.81%) for the ECH and from 0.67% to 1.52% (with an average 0.99%) for the WCH, showing an increasing trend with time (Figure 4a). This showed that there was no significant difference between ECH and WCH before the 1970s, while the TOC content at the WCH has been significantly higher than that at the ECH since the 1970s. The TN content showed a similar trend (Figure 4b), varying from 0.072% to 0.153% and from 0.086% to 0.259% for the ECH and WCH, respectively. The average content of TN at the WCH was about 1.28 times as that at the ECH since the 1860s, and increased to 1.43 times since the 1970s. There was a significant relationship between TOC and TN in both cores (R^2^ = 0.96, *p* < 0.01), indicating that the nitrogen was mainly in the form of organic nitrogen.

The vertical distribution of the C/N molar ratios at two sediment cores presented similar characters (Figure 4c), showing a decreasing trend from the bottom to the top for both cores. The ratios ranged from 6.79 to 9.93 and from 6.13 to 10.19 for the ECH and WCH, respectively. Unlike the TOC and TN content, the C/N ratio showed no significant difference between the ECH and WCH for both periods of pre-1970s and post-1970s.

The δ^13^C values of organic carbon in sediment ranged from −24.8 to −21.6‰ and from −24.5 to −21.7‰, with an average value of −23.4 and −23.1‰ for the ECH and WCH, respectively (Figure 4d). Both cores showed a depleting trend of δ^13^C values since the 1860s. The values of δ^15^N showed an opposite distribution character with an increasing trend from the bottom to the top (Figure 4e). The values at the ECH ranged from +2.07 to +7.57‰, with an average value of +5.13‰. The values at the WCH varied from +1.73 to +10.42‰, with an average of +5.69‰. For the values of δ^13^C and δ^15^N, no significant difference between the ECH and WCH was observed throughout the profiles.

### 3.3. Organic Carbon and Nitrogen Burial Rates

**Figure 5:**
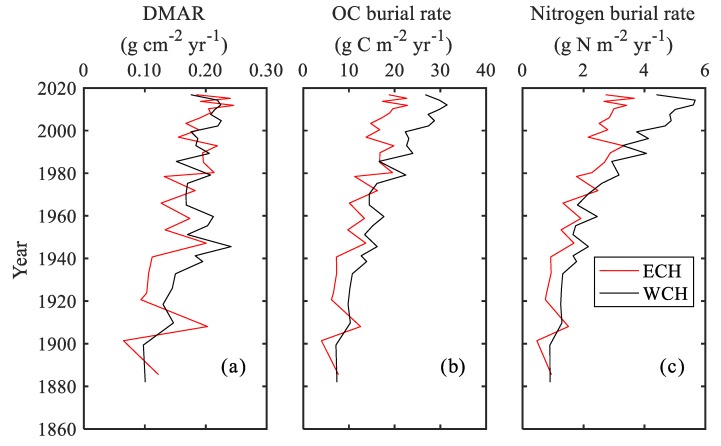
Distribution of (**a**) dry mass accumulation rates (DMAR), (**b**) organic carbon burial rates and (**c**) nitrogen burial rates in sediment at the WCH and ECH.

In Chaohu Lake, the dry mass accumulation rates (DMAR) generally showed a steady increasing trend from the 1860s to 1960s, ranging from 0.065 to 0.246 g cm^−2^ yr^−1^ (Figure 5a).

Since the 1960s, the rates showed no difference between the ECH and WCH, which oscillated between 0.127 and 0.246 g cm^−2^ yr^−1^, with average values of 0.191 and 0.194 g cm^−2^ yr^−1^ for the ECH and WCH, respectively. The temporal variations of carbon and nitrogen burial rates also presented increasing trends since the 1860s (Figure 5b,c). For organic carbon, the burial rates varied from 3.98 to 22.67 g C m^−2^ yr^−1^ and from 7.15 to 31.47 g C m^−2^ yr^−1^ for the ECH and WCH, respectively. The average rate in the WCH was 18.64 g C m^−2^ yr^−1^, which was 4.51 g C m^−2^ yr^−1^ higher than that in the ECH. However, the nitrogen burial rates were relatively lower. The rates varied from 0.47 to 3.67 g N m^−2^ yr^−1^ and 0.90 to 5.66 g N m^−2^ yr^−1^, with an average rate of 2.10 and 2.90 g N m^−2^ yr^−1^ for the ECH and WCH, respectively.

## 4. Discussion

### 4.1. Temporal Patterns of Organic Carbon and Nitrogen Burial in Chaohu Lake

**Figure 6:**
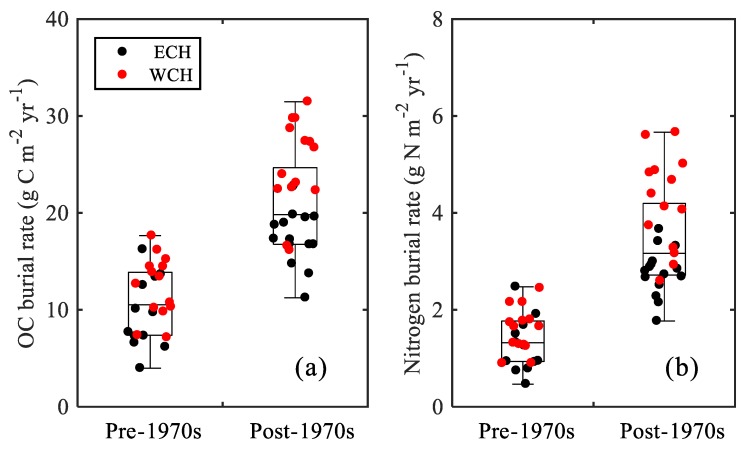
Boxplot of burial rates for the period of pre-1970s and post-1970s for (**a**) organic carbon and (**b**) nitrogen.

Lakes are considered to play a key role in the regional elements biogeochemical cycle at watershed, regional, and global scales, which are recognized as having vital roles in carbon and nitrogen sequestration, which buries considerable organic matter in the sediment [10,13,14,43,44,45]. There were significant increases in the carbon and nitrogen burial rates in Chaohu Lake (Figure 5b,c), suggesting an enhanced influence of human activities. Using one-way ANOVA to analyze the change in rates, there were significant differences between the period of the pre-1970s and the post-1970s (Figure 6a,b). The pre-1970s organic carbon burial rates were estimated as 8.95 ± 3.19 g C m^−2^ yr^−1^ for the ECH and 12.24 ± 3.71 g C m^−2^ yr^−1^ for the WCH. These estimations were close to the rates estimated by previous studies in Chaohu Lake [7,46,47] and lakes in the middle and lower reaches of the Changjiang River Basin [7,46]. Since the 1970s, the average burial rate was 17.68 ± 3.01 and 24.19 ± 5.27 g C m^−2^ yr^−1^ for the ECH and WCH, respectively, which are both about 1.98 times higher than the pre-1970s. Compared to the organic carbon burial rates in Dianchi Lake with 54.33 g C m^−2^ yr^−1^ [10], European lakes with ~60 g C m^−2^ yr^−1^ [14], and lakes in the USA with 46 g C m^−2^ yr^−1^ [5], the rates at Chaohu Lake were reasonably low. Nitrogen burial rates showed a similar temporal variation to carbon. The post-1970s nitrogen burial rates (2.76 ± 0.48 g N m^−2^ yr^−1^ for the ECH and 4.07 ± 1.07 g N m^−2^ yr^−1^ for the WCH) were about 2.43 and 2.63 times that for the pre-1970s (1.14 ± 0.44 g N m^−2^ yr^−1^ for the ECH and 1.55 ± 0.69 g N m^−2^ yr^−1^ for the WCH) for the ECH and WCH, respectively. The rates were close to that in Dianchi Lake with 1.02 g N m^−2^ yr^−1^ [10].

The distribution of carbon and nitrogen burial rates showed significant spatial heterogeneity in Chaohu Lake. In the pre-1970s period, despite the differences in the sedimentation rates between the ECH and WCH being relatively higher, there was no significant difference between the ECH and WCH for both the organic carbon and nitrogen burial rates (Figure 6a,b). This could be attributed to the close content of carbon and nitrogen in the sediment. Since the 1970s, the burial rates at the WCH were about 1.37 and 1.48 times that at the ECH for organic carbon and nitrogen, respectively. The DMAR between these two sites showed no difference (Figure 5a), while the contents of TOC and TN content in the western lake were significantly higher than that in the eastern lake since the 1970s (Figure 5a,b).This indicates that the site-dependent variability of burial rates was primarily driven by differences in the carbon and nitrogen contents. Since the 1970s, with rapid socio-economic development, nutrient loading originating from industrial wastewater, agriculture fertilization, and domestic sewage have been accelerated in the Chaohu Lake watershed [48]. This has led to a considerable amount of nutrients entering Chaohu Lake from the rivers [49], which first enter the western lake [50]. Such enrichment has significantly promoted TN, total phosphorus (TP) concentration and primary productivity in the overlying water in the western Chaohu Lake [51,52]. Besides this, the TP content in sediment varied from 0.400 to 0.533 mg P g^−1^ (with an average 0.449 mg P g^−1^) and 0.459 to 1.106 mg P g^−1^ (with an average 0.772 mg P g^−1^) for the ECH and WCH, respectively, showing an increasing trend from the bottom to the top for both cores (unpublished data). The storage of phosphorus could be released to the overlying water [52], which can promote the primary productivity and further promote the carbon and nitrogen burial rates in the sediment.

Although total phosphorus concentrations in the overlying water were higher than 100 μg L^−1^ throughout the year [51], the organic carbon burial rates in Chaohu Lake were relatively lower than those in European lakes [14] and American lakes [5]. This could be attributed to the high decomposition rate in the water column and in the sediment. For example, a study in the adjacent Taihu Lake, a hyper-eutrophic lake, showed that nearly 80% of organic matter could be decomposed within one month before sedimentation [53]. After sedimentation, organic matter decomposition was largely dependent on O_2_ exposure [8,14,16]. Chaohu Lake was marked as a typical shallow lake with no water column stratification throughout the year, resulting in organic matter in the top layers being exposed to oxygen and labile to decomposition.

### 4.2. Sources of Carbon and Nitrogen in Lake Sediment

**Figure 7:**
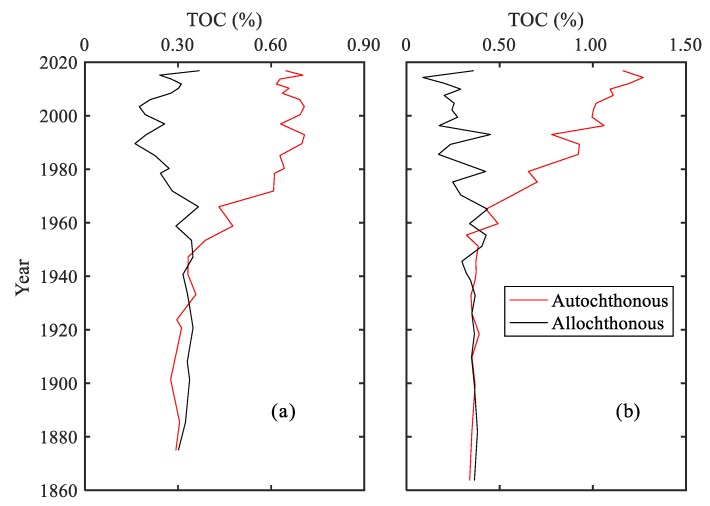
Variation of contribution from autochthonous and allochthonous organic matter at (**a**) the ECH and (**b**) WCH.

Organic matter in sediment is from a mixture of different sources. The atomic ratio of C/N in sediment has been widely utilized to identify the different sources of organic matter in lake sediments [29,54]. As the atomic ratios of C/N are characterized with distinctive values for autochthonous sources (3 to 8) and allochthonous sources (>20), the vertical variation of the C/N ratio can be used to reflect the change in the relative contribution proportion from these two sources [2,27]. A decrease of C/N with depth suggests that sediments receive a higher proportion from autochthonous sources. Conversely, an increase of C/N indicates a higher proportion from terrestrial organic matter input [29]. The C/N ratios at the ECH and WCH varied from 6.13 to 10.19 with a decreasing trend from the bottom to top (Figure 4c), which indicates an increasing organic matter proportion from autochthonous source. The binary mixing model, which was introduced by Qian [55], was applied to quantitatively identify the contribution of autochthonous and allochthonous organic matter sources. This model is a zero-order approximation model that uses the content of TOC and TN and C/N ratio to estimate the contribution of autochthonous and allochthonous organic carbon and has been widely used in source analysis [40,47,56]. The results showed that the organic matter originating from these two sources was close for both the ECH and WCH before the 1970s (Figure 7). The autochthonous source showed an increasing trend and became the dominant source since the 1970s, which is consistent with previous study in Chaohu Lake [47] and Taihu Lake [56]. The decreasing contribution from terrestrial sources could be attributed to the construction of reservoirs within the Chaohu Lake watershed. To ensure the security of drinking and irrigation water, a great number of reservoirs such as the Longhekou Reservoir and Dongpu Reservoir have been built since the 1950s, meaning that less terrestrial organic matter enters into the lake.

Sources of different organic matter are characterized by distinctive isotopic compositions. Values of δ^13^C in sediments have been widely used to identify organic matter sources and primary productivity in lakes [57,58]. Phytoplankton preferentially assimilates ^12^CO_2_ in water, which can be frequently exchanged with atmospheric CO_2_ [29,58]. With the increase in productivity, more ^12^CO_2_ can be incorporated in phytoplankton and leave the newly produced organic matter depleted in δ^13^C values. In Chaohu Lake, the values of δ^13^C ranged from −25.0 to −21.9‰, indicating that organic matter in sediment was a mixture of terrestrial plants and allochthonous phytoplankton. Prior to the 1970s, the values (−22.3 ± 0.40‰ for the ECH and −22.1 ± 0.80‰ for the WCH) were relatively enriched, suggesting a higher contribution from terrestrial sources. However, the δ^13^C values had presented a continuous decreasing trend with a depletion of 1.5 to 3.0‰ since the 1970s. This suggests that an increase of primary productivity in the lake has yielded a decrease of δ^13^C in organic matter and was further buried in sediment with increasing TOC content and a decreasing C/N ratio. In spite of this, the close δ^13^C values further indicated that organic matter sources were similar for the eastern and western Chaohu Lake.

Unlike carbon sources, the δ^15^N value in algae is more controlled by the isotopic compositions of DIN, which is highly influenced by human activities [59]. In Chaohu Lake, the δ^15^N values in the lower layers of the sediment cores were close, with an average value of +3.87 ± 1.07‰ and +3.42 ± 1.12‰ for the ECH and WCH, respectively. This indicates that nitrogen sources were constant during this period. Since the 1970s, the δ^15^N values have presented an increasing trend, which is consistent with the TOC and TN contents (Figure 4a,b,e). Nitrate from sewage and agricultural runoff are characterized by typically higher δ^15^N values [30]. The supply of ^15^N-rich nitrate can produce δ^15^N-enriched phytoplankton [59,60], which can further lead to the rise in δ^15^N values in sediment. In addition, the decrease in N_2_-fixing cyanobacteria due to the shift of ecological structure can also lead to risingδ^15^N values in lakes and organic matter in sediments [17].

Moreover, early diagenesis after sedimentation may have a potential influence on the isotopic composition of organic matter in sediment [60]. For the isotopes, light carbon (^12^C) and nitrogen (^14^N) in organic matter will preferentially mineralize during early diagenesis, potentially resulting in the enrichment of ^13^C and ^15^N. Previous study showed that early diagenesis-caused isotopic fractionation was under detection for δ^15^N in lake sediment [2]. In this study, the values of δ^13^C and δ^15^N were relatively stable in the downward sediment layers (Figure 4d,e). This indicates that early diagenetic-caused isotopic change was insignificant in the sedimentary organic matter for Chaohu Lake. Furthermore, nitrogen in organic matter was preferentially degraded when compared to carbon, which could lead to the increase of the C/N ratio [26]. For the values before the 1970s, the C/N had no significant oscillation for both the ECH and WCH (Figure 4c), indicating that early diagenesis had no significant influence on the C/N ratio in Chaohu Lake. Therefore, the geochemical proxies of sediment can be effectively used to reflect environment change in Chaohu Lake.

### 4.3. Driving Factors of Carbon and Nitrogen Burial

**Figure 8:**
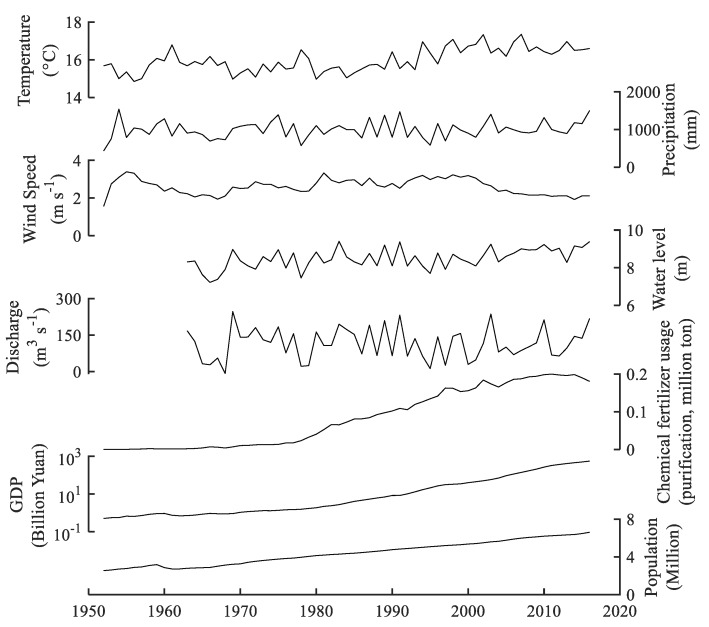
Temporal variation of air temperature, precipitation, wind speed, Chaohu Lake water level, Chaohu Dam discharge, chemical fertilizer usage, GDP, and human population in the Chaohu Lake watershed.

The increasing trend of carbon and nitrogen burial rates in lakes has been confirmed by previous studies, which are driven by natural and artificial factors [5,7,13,14,44]. Temperature is one of the most important driving factors. Rising temperature can lead to the promotion of nutrient release from sediments [61] and phytoplankton productivity in lakes [23]. Moreover, an increase in temperature can also accelerate organic matter decomposition and reduce burial rates in sediment [13,14,62,63]. This shows that temperature has a negative effect on organic carbon burial rates in sediment under experimental treatment conditions [15]. However, regional scale studies revealed that increasing temperature could lead to more carbon being buried in sediment [13]. The annual air temperature showed an increasing trend with 15.8 °C in 1953 to 16.6 °C in 2016 in Chaohu Lake (Figure 8). The air temperature showed a positive relationship with the carbon and nitrogen burial rates (Table 2). This suggests that warming-caused organic carbon and nitrogen burial was greater than the decomposition in sediment, and that warming was an important driving factor for the organic carbon and nitrogen burial rate in Chaohu Lake.

Precipitation has a positive influence on the organic carbon burial rates in lakes by carrying terrestrial organic carbon and flushing nutrients into lakes [64]. In the Chaohu Lake watershed, precipitation has shown no significant variation since the 1950s (Figure 8) and had a less direct influence on the carbon and nitrogen burial rates in the lake (Table 2). However, this does not mean that precipitation had no effect on the burial rates. Intensive human activities have a strong effect on the burial rates in sediment, which can weaken the effect of precipitation on the burial rates in lakes [14] and can make the effect not real [13]. Previous study has shown that precipitation has an important role in regulating the carbon burial rates in less disturbed watersheds by human activities [13]. Indeed, precipitation-controlled inter-annual and/or inner-annual drier and wetter conditions can greatly alter organic matter and nutrient export from the terrestrial system [25,28]. Intensive precipitation can flush a considerable quantity of nutrients into lakes [65,66], which can further promote primary production. Therefore, the change of temporal precipitation patterns can indirectly promote the burial rate of organic carbon and nitrogen in sediment.

Hydrological processes such as annual water level and hydraulic residence time are important factors in regulating nutrient dynamics in lakes [67,68]. Before impoundment in 1962, Chaohu Lake was a riverine lake that was characterized by high seasonal water-level fluctuation. After the construction of the Chaohu Dam in 1962, Chaohu Lake turned into a man-controlled reservoir with low water level fluctuation throughout the year. The change of water level had a direct influence on the ecological structure in the lake, leading to the disappearance of submerged vegetation and the appearance of algal bloom [37]. There was a significantly positive relationship of water level with the carbon and nitrogen burial rates in Chaohu Lake (Table 2), indicating that water level had an important role in controlling the organic carbon and nitrogen burial rate. The change of hydrological condition also led to a dramatic reduction in the annual water exchange volume between Chaohu Lake and the Changjiang River, which decreased from 13.6 × 10^8^ m^3^ to 1.6 × 10^8^ m^3^ [69]. This led to the enrichment of nutrients and a shift of ecological structure from a macrophytic-type lake to an algal type lake [36,37] and increased primary production in the lake since the 1970s.

Socio-economic development has also been an important controlling factor in organic carbon burial rates in lakes [7,13,44]. Population, GDP (gross domestic product), and chemical fertilizer usage have shown a significant positive correlation with carbon and nitrogen burial rates (Table 2). In the Chaohu Lake watershed, population and GDP have experienced a continuous increase during the last few decades (Figure 8), with about 2.54 million people and 0.51 billion Yuan in 1952 and 6.61 million people and 557.0 billion Yuan in 2016. To meet the needs of agricultural development, chemical fertilizer usage has shown a steady increasing trend before the 1970s and a rapidly increasing trend since the 1980s (Figure 8). As a result, a substantial quantity of nutrient originating from agricultural runoff, industrial wastewater and domestic sewage has been discharged into Chaohu Lake. In addition, human activities such as deforestation, cultivation pattern changes, mining, and urbanization could be contributors to nutrient export. The enrichment of nutrients has consequently promoted primary production in the lake as well as organic carbon and nitrogen burial rates in sediment.

The results of the PCA show that the first and second principal component (PC) can explain a large extent of variance of the driving factors (over 99.9%). PC1 is most related to chemical fertilizer usage (Table 3), which indicates that chemical fertilizer usage was the predominant driving factor for carbon and nitrogen burial in Chaohu Lake. PC2 is associated with precipitation and discharge of the Chaohu Dam, which represents a natural driving force. The positive coefficients mean that an increase of precipitation can result in more organic carbon and nitrogen burial in sediment, which can be referred to the enhanced nutrient amount from the terrestrial ecosystem. Further, PC1 and PC2 was used to establish a multiple linear regression with organic carbon and nitrogen burial rates. The equations for organic carbon can be expressed as:

y = 15.10 + 0.0000292 PC1 + 0.00404 PC2 R^2^ = 0.48, *p* = 0.0102 (7)

y = 23.03 + 0.0000799 PC1 − 0.00133 PC2 R^2^ = 0.88, *p* < 0.0001 (8)

for ECH and WCH, respectively. For nitrogen:

y = 2.69 + 0.00000487 PC1 + 0.000380 PC2 R^2^ = 0.47, *p* = 0.0110 (9)

y = 3.96 + 0.0000141 PC1 − 0.000201 PC2 R^2^ = 0.87, *p* < 0.0001 (10)

for ECH and WCH, respectively, where, y is the burial rate of organic carbon or nitrogen, PC1 is the first principal component, and PC2 is the second principal component. The regression relationship (R^2^) at WCH is higher than at ECH, which can confirm that the western Chaohu Lake is more susceptible to environmental change than the eastern Chaohu Lake.

## 5. Conclusions

Geochemical proxies in sediments were used to analyze the temporal changes of organic carbon and nitrogen burial rates as well as the driving factors over the last 150 years in Chaohu Lake. The results showed that carbon and nitrogen burial rates varied from 3.98 to 31.47 g C m^−2^ yr^−1^ and from 0.47 to 5.66 g N m^−2^ yr^−1^, respectively. The burial rates in the western lake were higher than those in the eastern lake. The average burial rates of the entire lake after the 1970s were about 1.98 and 2.53 times higher than the rates before the 1970s for carbon and nitrogen, respectively, indicating accelerated burial rates. The increase in δ^13^C values and a decrease in the C/N ratio indicated that primary productivity in the lake was the main organic matter source, and the increasing δ^15^N values suggest enhanced nutrient loading from Hefei City since the 1970s. The increase of organic carbon and nitrogen burial rates was positively dependent on climate factors and socio-economic development, which indicates that accelerating rates of organic carbon and nitrogen will accumulate in the Chaohu Lake sediment in the future.

## Figures and Tables

**Table 1:** Hydrological and chemical parameters at the sampling sites. DO: dissolved oxygen; TN: total nitrogen; TP: total phosphorus.

Features ^1^	ECH	WCH
Core location	117°36′14.38″ E, 31°31′28.92″ N	117°22′20.49″ E, 31°37′50.59″ N
Sediment core length (cm)	28	29
Water depth (m)	3.5	2.5
Water temperature (°C)	21.0	20.0
DO (mg L^−1^)	7.27	7.88
TP (mg P L^−1^)	0.117	0.144
TN (mg N L^−1^)	1.51	2.95
NO_3_^−^ (mg N L^−1^)	0.90	2.31
NH_4_^+^ (mg N L^−1^)	0.050	0.073
PO_4_^3−^ (mg P L^−1^)	0.032	0.047
Chl a (mg L^−1^)	0.0252	0.0436

^1^ The characteristics are the parameters of overlying water at ECH and WCH.

**Table 2:** Correlation coefficients between the variables and organic carbon and nitrogen burial rates.

Driving Factors	ECH		WCH	
	OC Burial Rate	Nitrogen Burial Rate	OC Burial Rate	Nitrogen Burial Rate
Temperature	0.47 *	0.42	0.82 **	0.82 **
Precipitation	0.56 *	0.58 *	0.28	0.22
Wind speed	−0.29	−0.19	−0.30	−0.33
Water level	0.72 **	0.70 **	0.77 **	0.72 **
Discharge	0.27	0.37	0.01	−0.08
Chemical Fertilizer usage	0.73 **	0.72 **	0.95 **	0.94 **
GDP	0.61 *	0.53 *	0.61 *	0.65 **
Population	0.79 **	0.73 **	0.96 **	0.95 **

** Correlation is significant at the level of 0.01. * Correlation is significant at the level of 0.05.

**Table 3:** The principal component coefficients for driving factors.

Factors	PC1	PC2	PC3	PC4	PC5	PC6	PC7	PC8
Temperature	6.08 × 10^−6^	−8.45 × 10^−4^	−3.87 × 10^−3^	−3.99 × 10^−1^	8.48 × 10^−1^	3.41 × 10^−1^	−6.02 × 10^−2^	−3.37 × 10^−2^
Precipitation	4.65 × 10^−4^	9.67 × 10^−1^	−2.55 × 10^−1^	9.31 × 10^−4^	5.12 × 10^−4^	−7.23 × 10^−4^	−2.51 × 10^−4^	−7.54 × 10^−5^
Wind speed	−2.75 × 10^−7^	−2.05 × 10^−5^	2.28 × 10^−3^	8.22 × 10^−1^	2.28 × 10^−1^	3.99 × 10^−1^	−1.37 × 10^−1^	3.07 × 10^−1^
Water level	3.23 × 10^−6^	1.73 × 10^−3^	2.95 × 10^−3^	−1.65 × 10^−1^	−2.64 × 10^−1^	6.06 × 10^−1^	7.13 × 10^−1^	1.67 × 10^−1^
Discharge	−3.36 × 10^−6^	2.55 × 10^−1^	9.67 × 10^−1^	−3.29 × 10^−3^	3.28 × 10^−3^	−1.02 × 10^−3^	−3.10 × 10^−3^	−8.57 × 10^−4^
Chemical fertilizer usage	1.00 × 10^0^	−4.49 × 10^−4^	1.27 × 10^−4^	8.85 × 10^−6^	3.00 × 10^−6^	−1.33 × 10^−5^	1.12 × 10^−5^	−3.80 × 10^−6^
GDP	1.24 × 10^−5^	1.63 × 10^−4^	−1.09 × 10^−3^	−3.54 × 10^−1^	−1.89 × 10^−1^	6.75 × 10^−2^	−4.01 × 10^−1^	8.21 × 10^−1^
Population	1.53 × 10^−5^	3.18 × 10^−4^	−8.48 × 10^−4^	−1.15 × 10^−1^	−3.50 × 10^−1^	5.94 × 10^−1^	−5.55 × 10^−1^	−4.51 × 10^−1^
